# Hydration-Dependent Thermal and Microstructural Characterization of a Collagen-Based 3D Matrix for Periodontal Regeneration

**DOI:** 10.3390/jfb17070318

**Published:** 2026-07-01

**Authors:** Cristian Cojocaru, Dragos Ioan Virvescu, Stefan Lucian Toma, Florinel Cosmin Bida, Gabriel Rotundu, Andrei Georgescu, Dana Gabriela Budala, Ioana Vata, Daniela-Lucia Chicet, Nicoleta-Monica Lohan, Monica Tatarciuc, Ionut Luchian

**Affiliations:** 1 Grigore T. Popa University of Medicine and Pharmacy Iasi-Romania, 700115 Iași, Romania; 2 Faculty of Materials Science and Engineering, “Gheorghe Asachi” Technical University of Iași, Blvd. Dimi-trie Mangeron 71A, 700050 Iași, Romania; 3 Faculty of Medicine, Apollonia University, Pacurari Street 11, 700511 Iași, Romania

**Keywords:** collagen scaffold, differential scanning calorimetry (DSC), scanning electron microscopy (SEM), thermal behavior, Mucoderm

## Abstract

**Background:** Collagen-based scaffolds are widely used in regenerative applications, where their structural organization and physicochemical stability are essential for clinical performance. This study aimed to evaluate the influence of hydration on the thermal behavior and microstructural characteristics of a collagen-based matrix (Mucoderm^®^, Botiss Biomaterial GmbH, Zossen-Germany). **Methods:** Differential scanning calorimetry (DSC) was used to investigate thermal transitions in dry and rehydrated samples, while scanning electron microscopy (SEM) coupled with energy-dispersive spectroscopy (EDS) was employed to assess surface morphology and elemental composition; **Results:** The dry sample exhibited a broad endothermic transition at 87.8 °C, which shifted to higher temperatures upon rehydration, reaching 103.5 °C and 112.4 °C after 15 and 30 min, respectively. A corresponding increase in enthalpy values was also observed. SEM analysis revealed a heterogeneous surface morphology characterized by alternating compact and less dense regions, while EDS confirmed the predominance of carbon and oxygen with minor elements present in trace amounts; **Conclusions:** These findings indicate that hydration influences both the thermal response and structural organization of the scaffold, highlighting the role of water–matrix interactions in determining its physicochemical behavior.

## 1. Introduction

Dental biomaterials, particularly those with regenerative or biomimetic potential, are frequently exposed to variations in hydration that can significantly influence their physicochemical properties and clinical performance. Thermal stability and phase transition behavior represent key parameters for understanding material performance in the oral environment, which is characterized by fluctuating temperature and moisture conditions [1].

In addition to these environmental challenges, dental biomaterials must maintain structural integrity and functional performance under complex mechanical and biological stresses. Repeated cycles of hydration and dehydration, combined with thermal fluctuations caused by food intake and oral habits, may induce microstructural changes, affecting material durability, dimensional stability, and interfacial adhesion to dental tissues [2]. These factors are particularly relevant for materials used in direct contact with soft or hard tissues, where long-term stability is essential for clinical success.

Recent advances in biomaterials science have focused on the development of materials with improved resistance to environmental stressors, including hydrogels, fiber-reinforced composites, and bioactive materials capable of mimicking natural tissue behavior [3]. However, despite these developments, the relationship between hydration dynamics, thermal transitions, and clinical performance remains insufficiently explored, particularly under conditions that simulate the oral environment [4,5]. Understanding these interactions is critical for optimizing material selection and predicting long-term behavior in clinical applications.

Collagen-based membranes represent a cornerstone in contemporary oral regenerative procedures, being extensively used in guided tissue regeneration (GTR) and guided bone regeneration (GBR) due to their biocompatibility, biodegradability, and structural similarity to the native extracellular matrix [6]. Their clinical success relies not only on biological compatibility but also on the ability to maintain structural integrity and barrier function under the dynamic conditions of the oral environment [7,8].

In vivo, collagen membranes are subjected to continuous variations in hydration, temperature, and enzymatic activity, which may significantly affect their physicochemical properties and degradation behavior. Water plays a fundamental role in the structural organization and functional behavior of collagen-based biomaterials. Hydration influences intermolecular hydrogen bonding, fibrillar organization, dimensional stability, elasticity, and degradation kinetics [9]. Previous studies have demonstrated that variations in water content can significantly alter collagen denaturation temperature, thermal transitions, and mechanical resistance [10,11]. Since collagen membranes are routinely hydrated before clinical application, hydration-dependent modifications may directly affect membrane handling, structural integrity, and long-term barrier function during tissue healing [12].

At the same time, thermal fluctuations and moisture dynamics can alter collagen fibrillar organization and crosslinking stability, potentially leading to premature resorption or compromised barrier function [13]. Repeated hydration–dehydration cycles may induce modifications in intermolecular bonding, fibril packing density, and matrix cohesion, thereby influencing both the mechanical integrity and degradation kinetics of collagen-based scaffolds [14]. Since these materials are routinely exposed to saliva, temperature variations, and enzymatic activity in the oral cavity, understanding the physicochemical effects of hydration becomes particularly relevant for predicting long-term clinical performance [15,16]. Moreover, hydration-related structural modifications may affect membrane flexibility, handling properties, permeability, and tissue adaptation during regenerative procedures, ultimately influencing healing outcomes and regenerative predictability.

Differential scanning calorimetry (DSC) is a well-established technique for the investigation of thermal properties of biomaterials, providing valuable information regarding thermal transitions, endothermic and exothermic processes, and structural changes induced by external factors such as hydration [13,17]. In water-sensitive materials, rehydration can alter molecular organization, polymer chain mobility, and intermolecular interactions, thereby directly affecting thermal behavior [18,19]. In collagen-based systems, water molecules actively participate in stabilizing the triple-helical structure through hydrogen bonding and modulation of intermolecular spacing. Consequently, variations in hydration state may significantly influence denaturation temperature, thermal stability, and the energy required for structural disruption. DSC analysis therefore represents a valuable method for investigating hydration-dependent physicochemical modifications and for evaluating the stability of biomaterials intended for regenerative applications.

In this context, the comparative evaluation of dry and rehydrated samples facilitates the assessment of water-induced modifications in thermal stability and structural organization. Additionally, scanning electron microscopy (SEM) provides essential insights into surface morphology and structural changes, enabling a comprehensive correlation between microstructure and thermal properties [20].

Despite the widespread clinical use of collagen scaffolds, limited information is currently available regarding the direct correlation between hydration state, thermal behavior, and microstructural organization under controlled experimental conditions. Most available investigations focus predominantly on biological outcomes or mechanical performance, while the combined evaluation of hydration-induced thermal transitions and morphological characteristics remains insufficiently explored. Therefore, a more integrated characterization approach may contribute to a better understanding of scaffold behavior and improve the predictability of regenerative biomaterials in clinical practice.

Therefore, the aim of this study was to investigate the effect of rehydration on the thermal behavior of the samples using DSC and to correlate these findings with morphological characteristics assessed by SEM.

## 2. Materials and Methods

To investigate the effect of hydration on the thermal and structural properties of the analyzed material, a combined experimental approach was employed, integrating differential scanning calorimetry (DSC) and scanning electron microscopy (SEM).

This methodological framework enabled a comprehensive evaluation of both thermal behavior and surface morphology, facilitating the correlation between structural features and physicochemical properties.

### 2.1. Materials

The 30 analyzed samples of Mucoderm^®^ (Botiss Biomaterial GmbH, Zossen, Germany) were included in solid form with a spherical geometry and a diameter of 10 mm and were investigated both in the dry state and after rehydration in distilled water.

The choice of a spherical shape was intended to ensure a uniform distribution of mass and thermal gradients during DSC analysis, minimizing edge effects and allowing a more homogeneous heat transfer throughout the sample. In addition, the controlled diameter facilitated reproducibility of the experimental conditions and ensured consistency between measurements.

Mucoderm^®^ (Botiss Biomaterials GmbH, Zossen, Germany) is a commercially available porcine-derived acellular collagen matrix obtained through a multi-stage purification process designed to remove all non-collagenous proteins, cellular components, and potential microbial contaminants [21].

The manufacturing process yields a three-dimensional stable matrix composed of naturally crosslinked type I and type III collagen fibers, preserving the native extracellular matrix architecture of porcine dermis [21]. Unlike flat collagen membranes used exclusively as barrier devices, Mucoderm^®^ presents an open porous collagen network with interconnected pore spaces that facilitate cell adhesion, migration, and vascularization upon implantation [22]. The three-dimensional character of this matrix is defined not by self-fabrication, but by its volume-occupying porous architecture, which supports tissue ingrowth throughout its entire thickness, distinguishing it structurally from conventional flat barrier membranes.

Synchrotron-based X-ray tomographic microscopy has previously confirmed the three-dimensional porous organization of this matrix at the microstructural level. Histological analyses further revealed that Mucoderm^®^ contains pre-existing vessel channel skeletons within its thicker dermal structure, a feature that contributes to early vascularization following implantation [23].

These structural characteristics justify the use of the term “three-dimensional scaffold” in the context of the present study, referring to the spatial organization and tissue-engineering function of the material rather than implying custom fabrication.

The microstructural analysis was performed using a Quattro scanning electron microscope (Thermo Fisher Scientific, Waltham, MA, USA), operated in low vacuum mode (LVD) with secondary electron (SE) detection, at an accelerating voltage of 20.00 kV, a spot size of 4.0, and a working distance of approximately 9.3–9.6 mm. Images were acquired at magnifications of 100× and 400×. Elemental microanalysis was performed using an EDX detector (Super Xerophy, S-817XI, Horiba Stec, Kyoto, Japan).

Distilled water was selected as the hydration medium to ensure standardized and reproducible conditions across all experimental groups, eliminating the confounding effects of ionic strength, pH variation, and protein content that would be introduced by physiological fluids. This approach is consistent with established protocols for DSC characterization of collagen-based biomaterials, in which distilled or deionized water is routinely used to assess intrinsic hydration-dependent thermal properties under controlled conditions.

From a hydration perspective, the defined geometry also facilitates a more predictable diffusion of water within the sample, enabling a controlled comparison between different rehydration intervals.

To evaluate the influence of hydration degree on thermal properties, samples were rehydrated for controlled time intervals of 15 and 30 min at room temperature.

The two hydration time points of 15 and 30 min were selected to reflect the clinically relevant rehydration window recommended by the manufacturer for Mucoderm^®^ prior to surgical application, which specifies rehydration for five to twenty minutes depending on the technique and desired flexibility. The 30 min interval was additionally included to assess whether prolonged rehydration beyond the recommended clinical window induces further thermally detectable structural changes. This approach was designed to capture the transition from partial to more advanced hydration within a timeframe directly relevant to intraoperative handling, rather than to characterize the full kinetic hydration profile of the material.

A total of 30 samples were obtained from the same commercial batch of Mucoderm^®^ (Botiss Biomaterials GmbH, Zossen, Germany) to ensure material homogeneity and minimize inter-batch variability. Samples were allocated into three experimental groups according to hydration condition: ten samples in the dry state (Group 1), ten samples rehydrated for 15 min (Group 2), and ten samples rehydrated for 30 min (Group 3). Within each group, measurements were performed as technical replicates, meaning that multiple specimens from the same batch were analyzed independently under identical experimental conditions to assess the reproducibility of the thermal and morphological responses. The specimens were used to confirm the reproducibility of the observed thermal response under identical experimental conditions. Representative DSC thermograms and characteristic thermal parameters are reported for each hydration condition. Because the study was designed as a physicochemical characterization of the material rather than as a comparative statistical study, no inferential statistical analysis was performed. The reported thermal parameters (T_peak and ΔH) reflect values derived from the full set of measurements within each group.

### 2.2. Differential Scanning Calorimetry (DSC)

The thermal behavior of the samples in both dry and rehydrated states was investigated using differential scanning calorimetry (DSC). Measurements were performed using a NETZSCH DSC 200 F3 Maia (Netzsch, Selb, Germany), calibrated with standard reference materials (Hg, Bi, In, Sn, and Zn). To evaluate the influence of hydration on the thermal response, two rehydration conditions were considered: samples immersed in distilled water for 15 min and 30 min, respectively. After rehydration, excess surface water was gently removed prior to thermal analysis.

Sample fragments with masses of approximately 3.5 mg (dry sample), 7.5 mg (15 min rehydrated sample), and 6.9 mg (30 min rehydrated sample) were subjected to a controlled thermal program consisting of heating from room temperature to 150 °C at a rate of 5 K/min, followed by natural cooling.

The upper temperature limit of 150 °C was selected to capture the full range of thermally relevant transitions occurring in collagen-based biomaterials in both hydrated and dehydrated states. While the physiological temperature of the oral environment does not exceed 37 °C, DSC measurements at elevated temperatures serve a fundamentally different purpose: they provide information on the intrinsic thermal stability, crosslinking density, and structural integrity of the collagen matrix, rather than simulating clinical conditions. In the partially or fully dehydrated state, collagen denaturation transitions shift substantially toward higher temperatures, with the triple helix-to-random coil transition occurring in the range of 80–150 °C, depending on the residual water content and degree of crosslinking.

Specifically, the broad endothermic peak observed around 100 °C in dehydrated collagen is associated with the departure of strongly hydrogen-bonded residual water responsible for stabilizing the triple helix conformation, while transitions above 120 °C reflect deeper conformational changes in the collagen macromolecule. These parameters are widely used as quality control indicators for collagen-based scaffolds, providing comparative data on structural preservation across different hydration states and processing conditions, independently of the intended physiological environment.

Samples were placed in aluminum crucibles (40 μL) sealed with perforated lids, while an empty crucible was used as reference. All measurements were performed under an inert argon atmosphere with a constant flow rate of 50 mL/min in order to prevent oxidative processes and ensure thermal stability during analysis.

The heating rate of 5 K/min was selected based on previous studies demonstrating that DSC heating conditions significantly influence the position, shape, and resolution of thermal transitions. Gao et al. reported that heating rates are one of the major experimental parameters affecting the accuracy of DSC measurements, with excessive heating rates potentially leading to peak broadening and displacement of thermal events [13].

Similarly, Kahwaji et al. emphasized that moderate heating rates improve the reliability of thermal property determination by reducing thermal gradients within the sample and allowing a more accurate characterization of endothermic transitions [17]. In collagen-based materials, Bozec and Odlyha successfully employed comparable DSC conditions to investigate thermal denaturation phenomena and structural stability [24]. Therefore, a heating rate of 5 K/min was considered appropriate to ensure reliable characterization of hydration-dependent thermal transitions while maintaining methodological consistency among all experimental groups.

Controlled inert atmosphere conditions were maintained throughout all analyses in order to reduce oxidative interference and improve reproducibility of the thermal measurements.

Data acquisition and processing were performed using Proteus software, version 9.0 (Netzsch, Selb, Germany), including baseline correction, peak integration, and determination of thermal transition parameters.

Although sample masses differed between experimental groups (3.5 mg for the dry sample, 7.5 mg for the 15 min rehydrated sample, and 6.9 mg for the 30 min rehydrated sample), all enthalpy values were automatically normalized to sample mass by the Proteus software (Netzsch, Selb, Germany) and expressed in J/g. Mass-normalized enthalpy values are therefore directly comparable across groups, regardless of absolute sample mass, as is standard practice in DSC analysis of collagen-based biomaterials.

Nevertheless, the increased mass observed after rehydration reflects greater water uptake by the collagen matrix. Consequently, the progressive increase in enthalpy should be interpreted not only in terms of hydration-dependent structural adaptations but also in relation to the higher amount of absorbed and bound water participating in the thermal transition.

### 2.3. Scanning Electron Microscopy (SEM)

Surface morphology of the samples was investigated using scanning electron microscopy (SEM) in order to evaluate the microstructural organization of the material. Prior to SEM examination, the specimens were sputter-coated with a gold layer approximately 10 nm thick using a Quorum Q150R ES sputter coater (Quorum Technologies Ltd., Lewes, UK) in order to improve surface conductivity and minimize charging effects during imaging. The observations were performed at different magnifications (100× and 400×) to assess both the global surface architecture and local microstructural features.

The magnifications selected were chosen to provide an overview of the macroporous architecture, surface morphology, and the alternating compact and less dense regions characteristic of the Mucoderm^®^ matrix across all three hydration states. At these magnifications, the overall three-dimensional porous organization, pore interconnectivity, and hydration-induced structural changes at the tissue level can be reliably assessed, consistent with approaches reported in the SEM characterization of comparable porcine-derived collagen matrices.

The primary objective of the SEM analysis in the present study was to correlate macrostructural morphological changes with the thermal transitions identified by DSC, rather than to resolve nanoscale fibrillar organization or D-banding patterns of individual collagen fibrils, which would require higher magnifications or complementary techniques such as transmission electron microscopy or atomic force microscopy.

SEM analysis enabled the identification of structural heterogeneity, including the coexistence of dense and less compact regions, as well as the evaluation of surface continuity and the absence of macroscopic defects.

The combination of SEM and EDS analysis enabled simultaneous evaluation of surface morphology and elemental distribution, facilitating the correlation between microstructural organization and physicochemical composition. This integrated approach is particularly relevant for collagen-based biomaterials, where structural heterogeneity can influence hydration behavior and thermal response.

Although collagen is known to contain nitrogen as a constituent element of its amino acid backbone (primarily in peptide bonds and in the amino acids proline and hydroxyproline), nitrogen was not reported as a prominent peak in the EDS spectrum. This is attributable to well-documented physical limitations of energy-dispersive X-ray spectroscopy in detecting light elements, particularly nitrogen (atomic number 7), whose fcharacteristic Kα X-ray emission line occurs at 0.392 keV. At this low energy, the nitrogen signal is inherently weak and subject to partial overlap with the carbon Kα line (0.277 keV) and the oxygen Kα line (0.525 keV), resulting in poor spectral resolution and low detection sensitivity under standard EDS operating conditions.

Furthermore, the detection efficiency for light elements such as carbon, nitrogen, and oxygen is strongly dependent on detector window type, accelerating voltage, and sample coating, and nitrogen signals at concentrations below approximately 5 wt.% may fall below the reliable detection threshold of conventional EDS systems.

The dominant carbon and oxygen signals observed in the EDS spectrum are consistent with the organic composition of the collagen matrix and are in accordance with EDS analyses of comparable collagen-based biomaterials reported in the literature.

Elemental microanalysis was performed by energy-dispersive X-ray spectroscopy (EDS) coupled to the scanning electron microscope in order to evaluate the elemental composition of the scaffold surface. Representative areas were selected from different regions of each specimen to ensure a comprehensive assessment of elemental distribution. Both qualitative spectra and elemental mapping analyses were acquired, allowing the identification of the major constituent elements and the evaluation of their spatial distribution throughout the analyzed surface.

The acquired spectra were used to determine the relative abundance of the detected elements, while elemental maps provided information regarding their homogeneity within the collagen matrix. Special attention was given to the distribution of carbon and oxygen, which represent the principal components of collagen-based biomaterials, as well as to the identification of minor elements present in trace amounts. The combined SEM–EDS approach enabled the correlation of microstructural observations with compositional characteristics and facilitated a more comprehensive characterization of the scaffold.

## 3. Results

**Figure 1:**
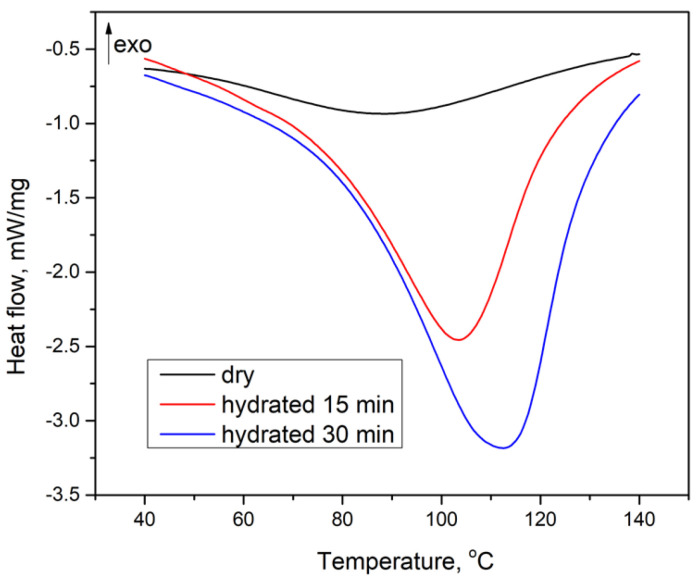
Variation in heat flow as a function of temperature for Mucoderm^®^ samples.

Differential scanning calorimetry (DSC) was used to evaluate the thermal behavior of the Mucoderm^®^ samples in dry and rehydrated states. The dry sample exhibited a broad endothermic transition illustrated in Figure 1, with a peak temperature (T_peak) of 87.8 °C and an enthalpy change (ΔH) of 219.7 J/g.

The DSC thermograms shown in Figure 1 are representative of the thermal response observed for each hydration condition. The corresponding thermal parameters are presented as characteristic values for each group and should be interpreted as descriptive physicochemical data rather than as mean values derived from inferential statistical analysis.

Rehydrated samples showed a similar thermal profile, with a progressively more pronounced endothermic peak. The peak temperature increased to 103.5 °C after 15 min of rehydration and to 112.4 °C after 30 min. A corresponding increase in enthalpy was observed, reaching 819.5 J/g and 1025.0 J/g, respectively, as seen also in Figure 1. This transformation can be associated with the thermal transition of the dry collagen matrix, influenced by residual water content and structural reorganization of collagen. This behavior is consistent with data reported in the literature for low-moisture collagen, where DSC transitions have been observed in the 80–100 °C range, depending on the degree of hydration and crosslinking.

Differential scanning calorimetry (DSC) was used to evaluate the thermal behavior of Mucoderm^®^ samples in dry and rehydrated states. The dry sample exhibited a broad endothermic transition with a peak temperature (Tpeak) of 87.8 °C and an enthalpy change (ΔH) of 219.7 J/g. Rehydration produced a progressive shift in the endothermic peak toward higher temperatures, reaching 103.5 °C after 15 min and 112.4 °C after 30 min. A corresponding increase in enthalpy was also observed, with values of 819.5 J/g and 1025.0 J/g, respectively. These findings indicate a hydration-dependent modification of the thermal response of the collagen matrix, with increasing water uptake associated with higher thermal transition temperatures and greater energy requirements. The DSC-derived thermal parameters are summarized in Table 1. All these values are summarized in Table 1.

**Figure 2:**
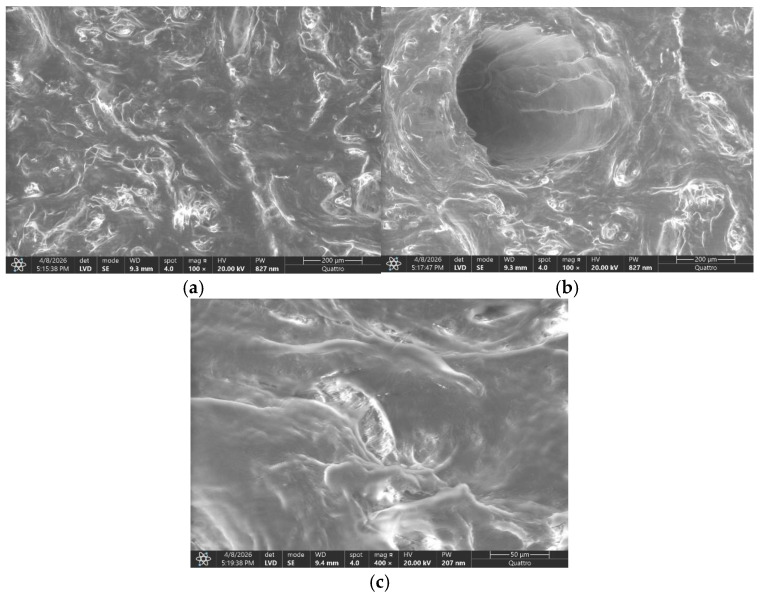
SEM morphology of the Mucoderm^®^ matrix: (**a**) general surface at 100×; (**b**) local variability at 100×; (**c**) microstructural detail of the selected area at 400×.

SEM observations revealed a continuous surface morphology characterized by the alternation of relatively compact regions and less dense areas at the micrometric scale as in Figure 2a,b. No macroscopic cracks or structural discontinuities were observed. At higher magnification (400×), the surface exhibited an irregular microstructural organization, with a granular–fibrillar appearance and localized variations in density as seen in Figure 2c.

**Figure 3:**
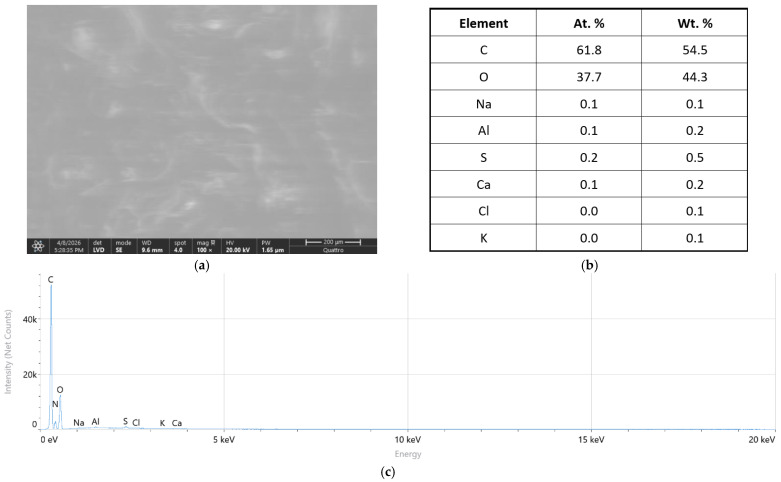
SEM–EDS analysis of the Mucoderm^®^ surface: (**a**) SEM image (100×); (**b**) elemental composition; (**c**) EDS spectrum.

Further SEM analysis indicated a relatively uniform surface at the micrometric level, with a fine texture and no evident large-scale defects in Figure 3a. Slight variations in contrast were observed, suggesting local differences in surface density or topography. EDS analysis confirmed the predominantly organic nature of the investigated scaffold, with spectra characterized mainly by carbon- and oxygen-related signals. Minor signals corresponding to additional elements were also detected in trace amounts. Due to the intrinsic limitations of EDS for the quantitative determination of light elements, the results should be interpreted primarily as a qualitative assessment of elemental composition rather than an accurate quantitative analysis as in Figure 3b. The corresponding EDS spectrum confirmed these findings, with dominant peaks associated with carbon and oxygen as seen in Figure 3c.

**Figure 4:**
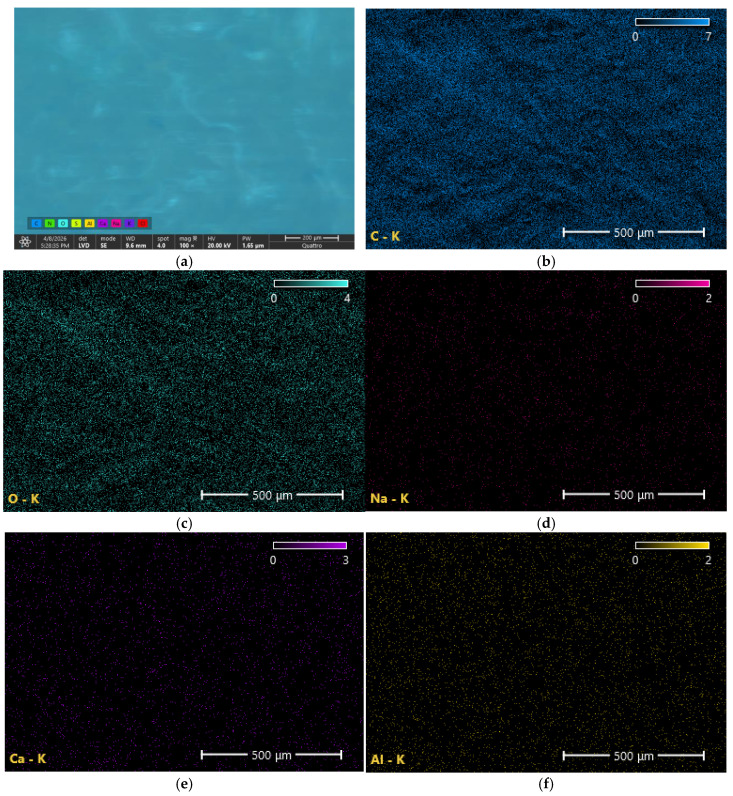
SEM–EDS analysis of elemental distribution in Mucoderm^®^: (**a**) SEM image; (**b**–**f**) elemental maps for C, O, Na, Ca, and Al.

The SEM image in Figure 4a reveals a continuous and relatively homogeneous surface at the micrometric scale, characterized by a fine texture, without major discontinuities or macroscopic defects. The reduced and uniform contrast suggests a compact and coherent structure of the material. Elemental distribution maps obtained by EDS in Figure 4b–f indicate a predominantly uniform distribution of the main elements. Carbon in Figure 4b and oxygen in Figure 4c show a homogeneous distribution across the entire analyzed surface, confirming the organic nature of the material.

## 4. Discussion

The DSC results demonstrate a clear and progressive influence of hydration on the thermal behavior of the Mucoderm^®^ collagen matrix. The dry sample exhibited a broad endothermic transition at 87.8 °C, characteristic of collagen with low moisture content, while rehydration for 15 and 30 min resulted in a progressive shift in the peak temperature to 103.5 °C and 112.4 °C, respectively, accompanied by a corresponding increase in enthalpy values. These findings indicate that water uptake enhances the thermal stability of the collagen network through hydrogen bond-mediated intermolecular interactions, which stabilize the triple-helix configuration and increase the energy required for thermal disruption.

The time-dependent nature of these changes suggests that water diffusion within the matrix is a gradual process, allowing progressive penetration into less accessible regions of the scaffold and promoting deeper structural reorganization with prolonged hydration.

Upon rehydration, a progressive shift in the endothermic peak toward higher temperatures was observed, reaching 103.5 °C and 112.4 °C after 15 and 30 min, respectively. This increase in T-peak suggests enhanced thermal stability of the collagen network in the presence of water. The absorbed water contributes to the stabilization of the triple-helix structure through hydrogen bonding and intermolecular interactions, requiring higher energy input for thermal disruption.

The marked increase in enthalpy values further supports this observation. The higher ΔH values recorded for rehydrated samples indicate a greater amount of energy required to induce the thermal transition, reflecting the contribution of both bound and absorbed water to the overall thermal response. This behavior is consistent with previously reported data for hydrated collagen systems, where water plays a key role in modulating structural organization and thermal properties.

Similar findings were reported by Schroepfer and Meyer [4], who demonstrated that increased hydration levels and crosslinking, modified the thermal transitions of bovine collagen matrices, resulting in higher denaturation temperatures and altered DSC profiles. Likewise, Zhang et al. [11] emphasized that hydration and intermolecular organization are major determinants of collagen thermal stability, particularly in structurally preserved collagen systems. The present results are consistent with these observations, supporting the concept that water-mediated stabilization mechanisms substantially influence collagen thermal behavior.

Comparison with other commercially available collagen membranes further supports the interpretation of the present findings. Tai et al. evaluated three widely used porcine collagen membranes (Bio-Gide^®^, Creos™ Xenoprotect, and Striate+™) and reported heterogeneous porous architectures characterized by alternating compact and less organized regions, together with comparable collagen fibril morphology among the investigated materials [25].

These observations are consistent with the SEM findings obtained in the present study, where the Mucoderm^®^ matrix also exhibited a heterogeneous microstructural organization with dense and less compact regions. Although direct comparisons should be interpreted cautiously because of differences in membrane origin, manufacturing procedures, and analytical protocols, the available evidence suggests that the structural features observed in Mucoderm^®^ fall within the range reported for clinically established collagen membranes used in guided tissue regeneration and guided bone regeneration procedures.

Furthermore, the hydration-dependent thermal modifications identified by DSC are consistent with the general behavior reported for collagen-based regenerative biomaterials, in which water uptake contributes significantly to the physicochemical stability of the collagen network.

From a clinical perspective, the observed hydration-dependent thermal modifications may have implications for the behavior of collagen membranes during clinical use. However, since mechanical performance and degradation kinetics were not directly investigated in the present study, any potential influence of hydration time on membrane handling characteristics, structural stability, or degradation behavior should be considered hypothetical. Further studies incorporating mechanical testing and enzymatic degradation analyses are necessary to determine whether the physicochemical changes identified by DSC translate into clinically relevant functional differences.

Comparable observations were described by Kasaj et al. [2], who reported that different rehydration protocols significantly influenced the biomechanical behavior of acellular collagen matrices used in oral regenerative procedures. Their findings suggested that hydration conditions may directly affect membrane flexibility, resistance to manipulation, and structural stability, which agrees with the hydration-dependent modifications identified in the present investigation.

The progressive nature of these changes, observed with increasing rehydration time, suggests that water diffusion within the matrix is not instantaneous but occurs gradually, leading to a time-dependent modification of the material’s internal structure. As a result, the thermal response becomes increasingly dominated by water–collagen interactions [20,26]. Consequently, hydration dynamics could directly affect membrane persistence and regenerative potential during the healing process [27,28,29].

In addition to hydrogen-bond-mediated stabilization, the progressive increase in Tpeak and ΔH observed after rehydration may also be associated with hydration-induced structural adaptations within the collagen matrix. Water uptake can promote fibril swelling, modify intermolecular spacing, and induce partial rearrangement of amorphous regions, thereby affecting molecular mobility and thermal behavior. Such phenomena may contribute to a more homogeneous distribution of water throughout the scaffold and increase the energy required for subsequent thermal transitions.

The differences observed between the 15 and 30 min hydration conditions suggest that water diffusion within the scaffold is a time-dependent process. The progressive increase in Tpeak and ΔH indicates continued hydration of the collagen matrix and a gradual modification of its thermal behavior with prolonged rehydration.

Differential scanning calorimetry studies have shown that the thermal transitions of collagen are strongly dependent on hydration state, as moisture content affects both the position and intensity of the endothermic peak [26]. It should be noted that DSC does not directly provide information regarding the secondary structure of collagen. Therefore, the present findings should be interpreted as evidence of hydration-dependent thermal and physicochemical modifications rather than direct proof of molecular conformational changes. Complementary spectroscopic techniques, such as Fourier-transform infrared spectroscopy, would be valuable for further elucidating potential alterations in collagen secondary structure induced by hydration.

In particular, variations in water content have been associated with changes in the stability of the collagen structure, as hydration alters intermolecular interactions and contributes to the organization of the fibrillar network [26,27]. This effect is consistent with the increase in energy required for thermal transition observed in the present study.

Furthermore, recent investigations emphasize that water is not only present as a removable phase but also plays an active role in defining the physicochemical behavior of collagen systems, influencing both structural cohesion and thermal response [1].

The SEM observations indicate that the Mucoderm collagen matrices exhibit a hierarchical structural organization. At the micrometric scale, the material is characterized by the alternation of relatively dense regions and more porous areas, reflecting heterogeneous architecture.

The heterogeneous microstructure identified by SEM may also contribute to differential water diffusion within the scaffold. Dense regions likely exhibit reduced permeability, whereas less compact areas may facilitate fluid penetration and local water retention. This structural variability could partially explain the progressive thermal modifications observed after rehydration and supports the concept that hydration dynamics are strongly dependent on scaffold architecture.

The coexistence of compact and less dense regions may additionally contribute to heterogeneous local mechanical behavior within the scaffold. More compact areas are likely to provide increased structural resistance, whereas porous regions may facilitate cellular infiltration and fluid exchange. This balance between stability and permeability represents an essential characteristic of collagen-based regenerative biomaterials and may contribute to improved tissue integration during periodontal healing.

The SEM observations performed at 100× and 400× magnifications demonstrated a heterogeneous surface morphology characterized by alternating compact and less dense regions, confirming the macroporous organization of the collagen scaffold. At the magnifications employed in the present study, SEM allowed the evaluation of overall surface morphology and structural heterogeneity but did not permit detailed visualization of individual collagen fibrils or pore wall ultrastructure. Therefore, the present observations should be interpreted as a characterization of scaffold architecture at the micrometric level. Higher-magnification SEM or complementary techniques, such as transmission electron microscopy (TEM) or atomic force microscopy (AFM), would be required for a more detailed ultrastructural evaluation.

Similar hierarchical structural arrangements have also been reported in other collagen-based extracellular matrices and regenerative biomaterials. Reznikov et al. [27] described collagen-containing biological structures as highly organized multi-scale systems in which nanoscale and microscale organization strongly influence functional behavior. In addition, Sun [29] emphasized that fibrillar collagen matrices exhibit complex mechanical responses directly associated with porous architecture and hydration-dependent structural interactions. These observations support the interpretation that the heterogeneous morphology identified in the present study may contribute to both mechanical adaptation and biological integration.

Elemental analysis further supports this interpretation. The predominance of carbon and oxygen, as revealed by EDS, confirms the organic nature of the material and is consistent with the expected composition of collagen-based matrices. The absence of marked concentrations of inorganic elements and the presence of only trace amounts of Na, Al, or Ca suggest that the material does not exhibit relevant contamination, and that these elements may be associated with processing or surface residues.

It should be noted that EDS analysis was primarily used as a qualitative tool for elemental characterization. The technique has recognized limitations regarding the accurate quantification of light elements such as carbon and oxygen, particularly in collagen-based materials. Therefore, the present EDS findings were interpreted mainly to confirm the organic composition and overall elemental homogeneity of the scaffold rather than to provide precise quantitative elemental measurements.

Despite the morphological variability observed in SEM images, the relatively uniform distribution of the main elements indicates that the material is chemically homogeneous at the micrometric scale. This combination of chemical uniformity and structural heterogeneity is a defining characteristic of biomimetic scaffolds.

The heterogeneous morphology observed by SEM, characterized by the coexistence of compact and less dense regions, reflects the intrinsic hierarchical organization of collagen-based materials. Such structural variability is consistent with previous reports describing collagen systems as multi-scale architectures, in which local differences arise from fibrillar organization rather than compositional changes [30,31].

The relatively uniform elemental distribution revealed by EDS supports this interpretation, indicating that the observed heterogeneity is mainly structural. This agrees with the current understanding of collagen-based biomaterials, in which functional behavior depends more on hierarchical organization and structural arrangement than on elemental variability [32].

This observation is also in agreement with the findings reported by Gonapinuwala et al. [20], who demonstrated that collagen materials may preserve substantial structural variability despite maintaining relatively homogeneous biochemical composition. Such behavior further supports the concept that functional performance in collagen-based scaffolds depends predominantly on hierarchical organization rather than elemental heterogeneity alone.

The present findings also support the importance of combining complementary characterization techniques when investigating collagen-based biomaterials. While DSC provides quantitative information regarding hydration-dependent thermal transitions, SEM and EDS contribute essential structural and compositional insights that cannot be obtained through thermal analysis alone [33]. The integration of these methodologies therefore enables a more comprehensive understanding of scaffold behavior and may improve the interpretation of biomaterial performance under clinically relevant conditions.

The present findings are also consistent with recent investigations emphasizing the importance of hydration in regulating the physicochemical and functional behavior of biomaterials exposed to oral conditions. Koehler et al. [15] demonstrated that temperature, moisture, and swelling dynamics significantly influence the stability and performance of oral biomaterials under simulated intraoral environments. Collectively, these observations reinforce the importance of considering hydration-dependent phenomena when evaluating collagen scaffolds intended for regenerative applications [34,35].

This study has several limitations that should be acknowledged. The experimental design was performed exclusively under controlled laboratory conditions and did not fully reproduce the complex biological environment of the oral cavity, including enzymatic degradation, cyclic mechanical loading, and long-term tissue interactions. Furthermore, only one commercially available collagen scaffold was investigated, limiting direct extrapolation to other biomaterials with different crosslinking patterns or structural organizations. In addition, no complementary spectroscopic analyses such as Fourier-transform infrared spectroscopy were performed. Therefore, potential hydration-induced modifications in the secondary structure of collagen could not be directly evaluated and should be investigated in future studies.

A limitation of the present study is the use of distilled water as the hydration medium, which does not fully replicate the complexity of the clinical oral environment, where collagen membranes are exposed to physiological saline, blood, saliva, and protein-containing tissue fluids. Ionic strength, pH, and the presence of plasma proteins are known to influence collagen hydration kinetics and may affect its thermal stability parameters as measured by DSC.

The use of distilled water was intentional, aimed at isolating the effect of hydration degree on the thermal behavior of the matrix under standardized conditions, thereby enabling reproducible inter-group comparisons. Future studies should investigate the thermal and structural behavior of Mucoderm^®^ following hydration in physiologically relevant media, including phosphate-buffered saline, simulated body fluid, or protein-enriched solutions, to better approximate in vivo conditions.

The present study examined two discrete hydration time points (15 and 30 min), which were selected based on the clinically recommended rehydration protocol for Mucoderm^®^. However, a comprehensive characterization of the hydration-dependent behavior of this matrix would benefit from a systematic assessment of water uptake, swelling ratio, and equilibrium water content across multiple time points, ranging from early rehydration (1–5 min) through to full equilibrium. Published data indicate that prolonged rehydration of Mucoderm^®^ beyond 30 min produces only minor additional effects on biomechanical properties [2], suggesting that the material approaches a near-equilibrium state within the timeframe examined in the present study. Nevertheless, gravimetric measurements of water uptake and swelling ratio at multiple time points would provide complementary quantitative data on hydration kinetics and should be incorporated in future investigations to fully characterize the hydration behavior of this collagen matrix.

A further limitation of the present study concerns the magnifications employed for SEM analysis (100× and 400×), which were selected to characterize macroporous architecture and hydration-induced morphological changes at the tissue level. These magnifications are insufficient to resolve nanoscale fibrillar organization, individual collagen fibril diameters, or the characteristic D-banding periodicity of collagen fibrils, which have been reported to require magnifications of 5000× to 20,000× or higher, as well as complementary techniques such as TEM and AFM. Future studies should incorporate higher-magnification SEM imaging to provide a more complete ultrastructural characterization of the Mucoderm^®^ matrix and to assess whether hydration-induced changes are also detectable at the fibrillar level.

Future investigations should include complementary mechanical analyses, extended hydration protocols, and in vivo validation models in order to better correlate physicochemical behavior with clinical performance.

Future research directions should also focus on the investigation of hydration-induced mechanical modifications in collagen scaffolds, particularly regarding tensile resistance, elasticity, compressive behavior, and fatigue stability under cyclic loading conditions. Since collagen membranes are continuously exposed to mechanical stress and moisture fluctuations in the oral environment, understanding the relationship between hydration dynamics and mechanical performance may contribute to improving the predictability of regenerative outcomes. In addition, further studies should evaluate the influence of hydration on cellular adhesion, fibroblast proliferation, angiogenic response, and enzymatic degradation under simulated periodontal and peri-implant conditions.

Another important aspect that warrants further investigation is the optimization of hydration protocols prior to clinical application. Variations in rehydration time, temperature, ionic composition, and storage conditions may significantly influence scaffold organization and functional behavior. Therefore, standardized hydration procedures could potentially improve membrane handling, structural stability, and long-term barrier function during regenerative therapy. Moreover, integrating advanced analytical approaches, including atomic force microscopy, micro-computed tomography, rheological analysis, and nanoindentation techniques, may provide a more detailed understanding of hydration-dependent structural adaptation in collagen-based biomaterials.

Future translational studies should additionally investigate the correlation between physicochemical properties and clinical outcomes in regenerative dentistry. Establishing direct relationships between hydration-dependent thermal behavior, structural organization, degradation kinetics, and tissue healing may facilitate the development of next-generation collagen scaffolds with improved functional stability and biological performance.

## 5. Conclusions

This study provides an integrated perspective on how hydration modulates the behavior of collagen-based scaffolds, by simultaneously linking thermal response with microstructural and elemental characteristics. Rather than being governed by compositional variability, the findings indicate that the performance of the material is controlled by its hierarchical structural organization and water–matrix interactions.

By combining DSC with SEM analysis, this work highlights the importance of multi-scale characterization in understanding the functional behavior of collagen scaffolds, an aspect that is often addressed only partially in the literature.

The results emphasize that even chemically homogeneous materials can exhibit complex structural and functional responses, driven by their internal organization.

These insights contribute to a more accurate framework for evaluating collagen-based biomaterials and support further investigation of hydration-dependent phenomena in regenerative applications. Although the present findings demonstrate marked effects of hydration on thermal behavior and microstructural organization, the potential influence of hydration time on membrane handling characteristics, degradation kinetics, and long-term clinical performance remains hypothetical and requires validation through dedicated mechanical and biological studies.

Future studies should further investigate the relationship between hydration-dependent thermal behavior and the long-term functional performance of collagen-based scaffolds under simulated oral conditions. In particular, integrating mechanical testing, degradation analysis, swelling kinetics, and biological evaluation may provide a more comprehensive understanding of scaffold stability and regenerative potential. Additionally, future research should explore the influence of enzymatic degradation, cyclic mechanical loading, and dynamic pH variations in order to better reproduce the complex oral environment.

Advanced multi-scale characterization approaches combining thermal, structural, and biological analyses may also contribute to the optimization of collagen-based biomaterials and support the development of more predictable regenerative strategies in periodontal and peri-implant therapy.

## Figures and Tables

**Table 1:** DSC-derived thermal parameters of Mucoderm^®^ samples in dry and rehydrated states.

Sample	Sample Mass [mg]	T Peak [°C]	DH [J/g]
Mucoderm^®^ dry	3.5	87.8	219.7
Mucoderm^®^ hydrated 15 min	7.5	103.5	819.5
Mucoderm^®^ hydrated 30 min	6.9	112.4	1025.0

## Data Availability

The data sets generated and/or analyzed during the current study are available from the corresponding author upon reasonable request.

## Abbreviations

DSC	Differential Scanning Calorimetry
SEM	Scanning Electron Microscopy
EDS	Energy-Dispersive Spectroscopy
GTR	guided tissue regeneration
GTB	Guided bone regeneration
ΔH	Enthalpy Change
Na	Sodium
Al	Aluminum
S	Sulfur
Cl	Chlorine
K	Potassium
Ca	Calcium
